# Reduction of Pathogens in Feces and Lymph Nodes Collected from Beef Cattle Fed *Lactobacillus salivarius* (L28), *Lactobacillus acidophilus* (NP51) and *Propionibacterium freudenreichii* (NP28), Commercially Available Direct-Fed Microbials

**DOI:** 10.3390/foods11233834

**Published:** 2022-11-28

**Authors:** Makenzie G. Flach, Onay B. Dogan, Wanda M. Kreikemeier, Kendra K. Nightingale, Mindy M. Brashears

**Affiliations:** 1 International Center for Food Industry Excellence, Department of Animal and Food Sciences, Texas Tech University, Lubbock, TX 79409, USA; 2 Livestock Logic, LLC, 79521 Rd 417, Callaway, NE 68825, USA

**Keywords:** *E. coli* O157:H7, *Salmonella*, *Clostridium perfringens*, enterobacteriaceae, peripheral lymph node, direct-fed microbials, pre-harvest food safety

## Abstract

The purpose of the study was to evaluate the prevalence and concentration of foodborne pathogens in the feces and peripheral lymph nodes (PLNs) of beef cattle when supplemented with direct-fed microbials (DFMs) in feedlots. Fecal samples were collected from the pen floors over a 5-month period at three different feedlots in a similar geographical location in Nebraska, where each feed yard represented a treatment group: (i.) control: no supplement, (ii.) Bovamine Defend: supplemented with NP51 and NP24 at a target dose of 9 log_10_CFU/g/head/day, and (iii.) Probicon: supplemented with L28 at a target dose of 6 log_10_CFU/g/head/day. Each fecal sample was tested for the prevalence of *E. coli* O157:H7 and *Salmonella*, and concentration of *E. coli* O157:H7, Enterobacteriaceae and *Clostridium perfringens*. Cattle were harvested and PLNs were collected on the harvest floor. Real-time *Salmonella* PCR assays were performed for each PLN sample to determine *Salmonella* presence. The cattle supplemented with both DFMs had reduced foodborne pathogens in fecal samples, but feces collected from the pens housing the cattle supplemented with Probicon consistently had significantly less *E. coli* O157:H7 and *Salmonella* prevalence as well as a lower *C. perfringens* concentration. While DFMs do not eliminate foodborne pathogens in fecal shedding and PLNs, the use of DFMs as a pre-harvest intervention allows for an effective way to target multiple pathogens reducing the public health risks and environmental dissemination from cattle.

## 1. Introduction

Food safety is a prominent concern for meat products in the United States. It is estimated that each year in the U.S., nine million people become ill, 56,000 are hospitalized, and 1300 die of foodborne diseases caused by known pathogens [1]. Of the 31 known pathogens, seven of the leading ones contribute to 90% of domestically acquired foodborne illnesses, hospitalizations, and deaths, causing about 112,000 disability adjusted life years (DALYs) annually in the U.S. [2]. “Healthy people” goals for 2030 include being free of preventable diseases, including foodborne diseases that originate from livestock [3]. While this 10-year goal may seem far reaching, these goals have driven research in identifying problem areas within the meat industry and in controlling and mitigating pre- and post-harvest contamination of products.

Naturally, livestock harbor a wide variety of microorganisms, some of which are identified as foodborne pathogens and have the potential to enter the food supply chain. Recent studies have identified *Escherichia coli* O157:H7, *Salmonella* spp., Enterobacteriaceae, and *Clostridium perfringens* all as common pathogens found in cattle feces [4,5,6,7,8,9,10,11]. In addition to causing foodborne illnesses, *C. perfringens* can result in animal health issues in cattle [12]. These pathogens from the live cattle can contaminate the carcass on the harvesting floor if proper dressing procedures are not sustained, which can lead to contamination in the final product, causing risk to the consumer. According to The Interagency Food Safety Analytics Collaboration (IFSAC), in 2019 beef contributed to an estimated 24.3% of foodborne *E. coli* O157:H7 infections and 6.2% of *Salmonella* infections [1]. While efforts should continue to focus on reducing these percentages, it is important to note that over the last seven years a declining trend in the percentage of illness contributed by beef, has been observed. In 2013, it was reported by IFSAC that beef contributed to 37.9% of *E. coli* O157:H7 illnesses and 9.1% of *Salmonella* illnesses [13]. This decline is greatly attributed to the significant efforts from the beef industry in terms of their advancement in pathogen reduction with the use of antimicrobials and emphasis on proper dressing procedures to prevent the contamination of carcasses [5]. While post-harvest interventions have been a main research focus over the years, these issues also warrant management strategies geared towards controlling the incoming pathogen loads entering commercial abattoirs on harvest-ready feedlot cattle.

Recent pre-harvest strategies have focused on the use of interventions, such as antimicrobials, bacteriophages, direct-fed microbials (DFMs), sodium chlorate, and vaccines [14,15,16]. The majority of the research on pre-harvest interventions in cattle is targeted to reduce the prevalence of *E. coli* O157:H7, mainly due to the recognition of cattle as the principal reservoir of *E. coli* O157:H7 [5,14,16,17,18,19]. However, cattle have a very complex natural gut microflora that colonizes multiple types of pathogenic bacteria; therefore, supplementing their diet with DFMs provides the ability to target multiple foodborne pathogens, including *E. coli* O157:H7, all within one intervention. Per the U.S. Food and Drug Administration, DFMs are defined as “products that contain live microorganisms like bacteria and/or yeast“ [20]. The use of DFM supplementation is an approach that may confer health benefits on the host while simultaneously reducing the colonization of pathogenic bacteria in the gastrointestinal tract [21]. The overall goal of feeding DFMs is to promote the growth of microorganisms that are competitive or hostile to pathogenic bacteria [5]. Recent studies have noted the efficacy of a DFM containing *Lactobacillus acidophilus* (NP51) and *Propionibacterium freudenreichii* (NP24), also commercially known as Bovamine^®^ Defend (CHR-Hansen; Hoersholm, Denmark) [17,22,23]. A systematic review from Sargent et al. reported that four of the five treatment comparisons evaluating a DFM combination of NP51 and NP24 achieved a significantly lower prevalence of *E. coli* O157:H7 in treated cattle, regardless of the dosage [16].

The bovine lymphatic system, which includes the peripheral lymph nodes (PLNs), has been identified as another potential source of pathogenic bacteria, more specifically *Salmonella*, contamination in ground beef [24,25,26]. Pathogen contamination of ground beef can occur when lymph nodes are incorporated in the manufacturing of beef trimmings. According to the Centers for Disease Control (CDC), *Salmonella* causes about 1.35 million infections, 26,500 hospitalizations, and 420 deaths in the United States every year [27]. Ground beef is an important vehicle for human exposure to *Salmonella*, and in recent years an increasing trend in outbreaks, due to this foodborne pathogen, has been seen [26]. Since 2017, three large *Salmonella* outbreaks have totaled nearly as many illnesses and more hospitalizations than all the *Salmonella* outbreaks linked to ground beef during the previous 36 years [28,29]. For this reason, in October 2019, the United States Department of Agriculture—Food Safety and Inspection Service (USDA-FSIS) announced the proposed standards for *Salmonella* in raw ground beef, allowing for a maximum of two positive samples in a 52-week period for a plant to be in “good standings” [30].

Significant efforts have been dedicated to surface area antimicrobial treatments in beef production and processing; however, harborage of *Salmonella* within the PLNs provides protection against these surface-oriented mitigation approaches [27]. This source of contamination would explain the greater prevalence of *Salmonella* observed in ground beef, relative to beef trim destined for ground beef. The effect of DFMs on *Salmonella* prevalence has not been widely studied; however, results from Vipham et al., 2014 illustrate that supplementing NP51 and NP24 in the diet of cattle may aid in reducing *Salmonella* prevalence and concentration in the subiliac lymph nodes [31]. Overall, the proposed *Salmonella* performance standards for ground beef will drive changes in the beef processing industry to utilize more effective *Salmonella* controls throughout the production line, such as the removal of lymph nodes from carcasses, and through pre-harvest interventions, such as DFMs, in the hope of reducing pathogen prevalence corresponding to the public health burden.

*Lactobacillus salivarius* is often isolated from animal and human samples and is known to produce bacteriocins and has been used as a probiotic supplement for animal and human health [32]. *Lactobacillus salivarius* L28 (Probicon) is a novel DFM isolated from ground beef samples. When compared to a variety of lactic acid bacteria strains in a screening study, L28 was shown to be more effective in inhibiting the growth of important foodborne pathogens, *Salmonella, E. coli* O157:H7 and *Listeria monocytogenes* [33]. Being a novel strain, its effectiveness in reducing foodborne pathogens in bovine feces in commercial conditions have not been extensively reported; however, preliminary data indicates that the use of L28 reduced and inhibited the growth of *E. coli* and *Salmonella* in artificially challenged cattle manure and had similar effects as a sub-therapeutic antibiotic in terms of gain performance and carcass traits in beef cattle (our unpublished data), indicating that it can be an alternative to reduce the dependence on antibiotics in the cattle industry.

The objectives of this study were to (i) evaluate the effect of supplementing DFMs in cattle’s diet compared to those who were fed a standard diet on the prevalence and/or concentration of *E. coli* O157:H7, *Salmonella*, Enterobacteriaceae, and *Clostridium perfringens* in the feces of beef cattle; (ii) evaluate whether the administration of Probicon (L28) [34] would have greater or similar effects to Bovamine Defend (NP51 and NP24); and (iii) evaluate the impact the supplementation of DFMs has on *Salmonella* prevalence in the peripheral lymph nodes of beef cattle.

## 2. Materials and Methods

### 2.1. Treatment Groups—Cattle Feed Yards

Three different cattle feed yards, all part of a Wagyu branded natural beef program, located in Eastern Nebraska and Western Iowa, were utilized in this study. The size of these three feed yards ranged from approximately 1000 to 9000 cattle on feed, with a range in pen sizes from approximately 75 to 300 head/pen. From each feed yard three pens were selected in which the same pens were sampled during every sampling event. The total number of cattle housed between the three pens sampled for each treatment group were approximately 300 head in the BD supplemented pens, approximately 600 head in the PC supplemented pens, and approximately 500 head in the control group pens. The cattle were on feed yard rations for a minimum of 200 days, when their diets consisted of high moisture corn, course roughage, corn silage, corn by-products, and supplements (no monensin or tylosin added). The study consisted of three treatment groups: (i.) Control: standard diet with no supplemented DFM; (ii.) Bovamine Defend (BD): standard diet supplemented with *Lactobacillus acidophilus* (NP51) and *Propionibacterium freudenreichii* (NP24) at a target dose of 9 log_10_CFU/head/day, which is a commercially available DFM (CHR-Hansen; Hoersholm, Denmark); and (iii.) Probicon (PC): standard diet supplemented with *Lactobacillus salivarius* (L28) at a target dose of 6 log_10_CFU/head/day, which is a commercially available DFM (NexGen Innovations LLC, Lubbock, TX, USA). DFMs were directly mixed with the daily feed using a commercial feed yard micro machine and distributed to the pens during the study period. The control yard had never been supplemented with any DFM or probiotic, the BD yard had been using BD for at least a year, and the PC yard had been on BD for at least five years but started using PC in March 2021.

### 2.2. Sample Collection

Fresh fecal samples from the pen floor were collected from a total of nine pens (*n* = 3 pens/feed yard) every 28 days over a five-month period, from May to September 2021. All three feed yards were sampled on the same day for each sampling event as outlined in Table 1. Fresh fecal material from the pen floors was collected using a teaspoon, one spoon per fecal pat, and placed into a sterile fecal specimen container. Approximately 5 to 10 samples were collected from each pen, depending on how many head were in the pen (<100 head = 5 samples; >200 head = 10 samples) to capture approximately 5% of the total animal population in the pens. A total of 300 fecal samples were collected among all treatments (Control: *n* = 110, BD: *n* = 75, PC: *n* = 115). Specimen containers containing fecal samples, were immediately chilled and shipped overnight to an independent, third-party laboratory.

Bovine subiliac PLNs were collected, over a two-month period, from the carcasses of the same cattle used in the fecal sampling portion of this study. Lymph nodes were immediately chilled and shipped overnight to the ICFIE Food Microbiology Laboratory at Texas Tech University for microbiological analysis. A total of 215 PLNs (control: *n* = 74, BD: *n* = 72, PC: *n* = 69) were collected between all treatment groups. All samples were collected from the carcasses of animals after post-mortem inspection at a large commercial USDA-FSIS inspected beef processing facility in Eastern Nebraska.

### 2.3. Fecal Sample Analysis

Fecal samples were blindly sent to a third-party laboratory (Food Safety Net Services, San Antonio, TX, USA) for analysis. Each fecal sample was homogenized manually in the original sample bag and 10 g sub-samples were transferred to a new, sterile, filtered sample bag for further processing for each analysis.

For the detection of *E. coli* O157:H7, the sub-samples were homogenized in 90 mL BAX MP Media (Hygiena, Camarillo, CA, USA) for 2 min and incubated at 42 °C for 18–24 h. After incubation, a 20 μL aliquot was placed into a BAX Cluster Tube filled with 200 μL of prepared BAX Lysis Reagent (150 μL protease + 12 mL lysis buffer). The tubes were heated at 37 °C for 20 min and at 95 °C for 10 min and then cooled to 2–8 °C for at least 5 min. After cooling, a 30 μL aliquot of lysate was placed into a BAX System Real-Time PCR Assay for the *E. coli* O157:H7 PCR tube and processed in a BAX Q7 Instrument for the BAX System Real-Time PCR Assay for *E. coli* O157:H7 Exact.

Only the positive *E. coli* O157:H7 samples were subjected to quantification by a modified three-tube most probable number method combined with molecular detection. Briefly, three replications of 10^−1^ to 10^−4^ dilutions of the retained original sample were placed in 96-well deep well plates and incubated at 42 °C for 18–24 h for enrichment. After incubation, 10 μL of enriched samples were added to 500 μL of pre-warmed (37 °C) Brain Heart Infusion (BHI) broth and incubated at 37 °C for 3 h and processed following the same steps for detection of *E. coli* O157:H7 using the BAX System Real-Time PCR Assay.

For *Salmonella* detection, 90 mL of buffered peptone water (BPW) was added to the bags, stomached for 2 min, and incubated at 35 °C for 18–24 h. After incubation, 5 μL aliquots were transferred to BAX Cluster Tubes filled with 200 μL prepared BAX Lysis Reagent (150 μL protease + 12 mL lysis buffer). The tubes were heated at 37 °C for 20 min, at 95 °C for 10 min, and cooled to 2–8 °C for at least 5 min. After cooling, 30 μL aliquots of lysate were transferred to BAX System PCR tubes and allowed to sit for 10–30 min in a cooling block to allow full hydration of the PCR pellets. After hydration, all tubes were placed in the BAX Q7 Instrument and the full process for BAX System Real-Time PCR Assay for *Salmonella* was run [35]. *Salmonella* in positive samples was not enumerated.

Enterobacteriaceae enumeration was conducted by serially diluting the sub-sample used for *Salmonella* detection in Butterfield’s Phosphate-Buffered Dilution Water (BPD) blanks and plating 1 mL on Petrifilm™ Enterobacteriaceae Count Plates (3M, St. Paul, MN, USA) [36]. The plates were incubated at 35 °C for 24 h and all red colonies with yellow zones and/or red colonies with gas bubbles with or without yellow zones were counted as Enterobacteriaceae.

For *C. perfringens* enumeration, the sub-sample used for *Salmonella* detection was serially diluted in BPD and 100 μL was spread plated on Tryptose Sulfite Cycloserine Agar and incubated anaerobically at 35 °C for 24 h. All black colonies were counted as *C. perfringens*.

### 2.4. Lymph Node Sample Processing and Salmonella Detection

Upon arrival, the surrounding fat and fascia were trimmed from the lymph node. Each PLN was weighed, submerged into boiling water for 3 to 5 s for surface sterilization, placed in a filtered Whirl-Pak bag (Nasco, St. Petersburg, FL, USA), and pulverized with a rubber mallet. Depending on the weight of the lymph node, either 20 mL (if PLN < 10 g) or 80 mL (if PLN 10–50 g) of BAX MP (Hygiena, LLC, Camarillo, CA, USA), a non-selective enrichment media, was aseptically added. The lymph node homogenate (LNH) was then stomached (Model 400 circulator, Seward, West Sussex, UK) at 230 RPM for 1 min. Following homogenization, the samples were incubated at 42 °C for 24 h.

After the desired incubation time was reached, a Real-Time (RT) *Salmonella* assay was performed on the pre-enriched samples for *Salmonella* prevalence using the Hygiena BAX^®^ Q7 system (Hygiena, LLC, Camarillo, CA, USA) according to the Association of Official Agriculture Chemists (AOAC) approved BAX^®^ System Q7 test protocol for RT *Salmonella* PCR assays following the procedure given in Section 2.3 [37].

### 2.5. Statistical Analysis

Longitudinal fecal contamination data was analyzed using the generalized estimating equations (GEE) approach using the “geepack” package available for R (Version 4.1.1.) [38]. The generalized estimating equation method is an extension to generalized linear models (GLM) that would allow the analysis of correlated data, such as repeated measures, longitudinal analysis, or nested designs, and continuous or discrete dependent variables. In this study, it was expected that the longitudinal design would require adjustments for the correlation within the sampled pens and both dichotomous and continuous outcomes were observed; therefore, GEE was selected as the method of statistical analysis [39,40]. Prevalence data were modeled using binomial distribution with logit link and concentration data were modeled using Gaussian distribution with identity link. The treatment type and the date of sampling were included in the models as categorical variables and the measurements were assumed to be nested within the different pens over repeated measures. The interaction terms were removed from the model if no significant interaction was detected by a preliminary analysis. All significant differences were evaluated using a *p*-value lower than 0.05. The results of the analysis were provided as a natural logarithm of the odds ratio (lnOR) for prevalence and the mean difference for concentration.

The PLN data was analyzed using R (Version 4.1.1) statistical software to evaluate the prevalence of *Salmonella* between the different treatment groups (control, BD, and PC). Contingency tables were produced for the prevalence of *Salmonella* (+/−). Within table differences were determined using Fisher’s exact test to determine the significance (*p* < 0.05) between treatment groups. Exact binomial confidence intervals for the prevalence estimates were calculated using the ‘propCI’ base function in R based on Clopper and Pearson [41].

## 3. Results

The results indicate that both DFMs used in the study are effective in reducing the prevalence and/or concentration of foodborne pathogens in cattle feces throughout the beef production chain. Overall, the feces samples collected from the pens housing cattle supplemented with Probicon had significantly less *E. coli* O157:H7 and *Salmonella* as well as *C. perfringens* concentration. *Salmonella* prevalence in PLNs collected from the cattle supplemented with DFM was lower compared to the presence of *Salmonella* in the control group. Although DFMs tested in this study and other similar studies do not eliminate the presence of pathogens [22,31,42,43,44], DFM supplements can reduce the public health risks and environmental dissemination from cattle.

### 3.1. Fecal Samples

#### 3.1.1. *E. coli* O157:H7 Prevalence and Concentration

**Figure 1:**
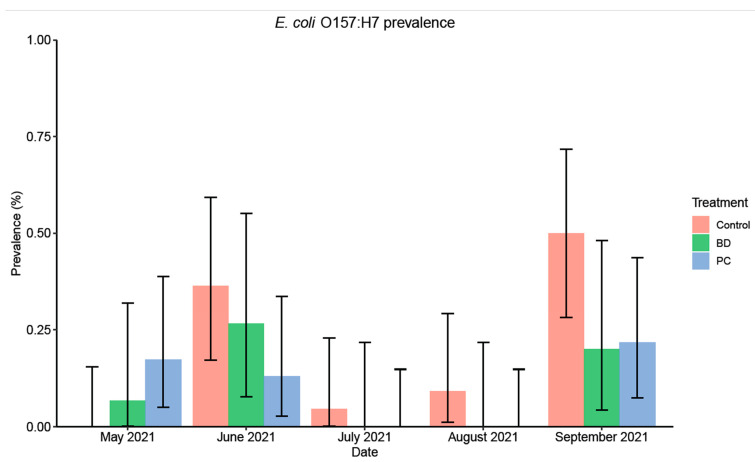
*E. coli* O157: H7 prevalence (%) of fecal samples collected from the pen floors over the course of a 5-month sampling period at three different feed yards (*n* = 3 pens/feed yard). Control (May: 0/22, June: 8/22, July: 1/22, August: 2/22, September: 11/22); BD (May: 1/15, June: 4/15, July: 0/15, August: 0/15, September: 3/15); PC (May: 4/23, June: 3/23, July: 0/23, August: 0/23, September: 5/23). Error bars represent 95% confidence intervals (CI).

The average *E. coli* O157:H7 prevalence was 20% (22/110), 10% (12/115), and 11% (8/75) for the control, PC, and BD groups, respectively, averaging over the sampling period. A comparison of *E. coli* O157:H7 prevalence at each sampling date for the two treatment groups and the control group is represented in Figure 1. At the beginning of the study period (May 2021), prior to any DFM supplementation, the prevalence in the fecal samples was 0.00 (95% CI: 0, 15) for the control group, 17% (95% CI: 5, 39) for the PC group, and 7% (95% CI: 0, 32) for the BD group. In order to account for prevalence differences in pens, the GEE analysis was nested within the different pens. At the end of the sampling period (September 2021), *E. coli* O157:H7 prevalence was higher than the initial prevalence (May 2021) for both the treatment and control groups, which could have occurred because prevalence typically increases in the summer months. Prevalence in the control group reached up to 50% (95% CI: 28, 72), while the PC and BD groups had a lower and more comparable prevalence of 22% (95% CI: 7, 44) and 20% (95% CI: 4, 48), respectively, and the difference was marginally significant (*p* < 0.10) when compared pairwise, possibly due to the limited sample size. Seasonal differences in prevalence were observed during the sampling period. Although the initial prevalence (May 2021) of the pens treated with PC was higher than the others, it was lower than the control and BD groups during sampling in June 2021. In July and August 2021, overall prevalence was low for all three groups combined. No *E. coli* O157:H7 was detected in the samples from the PC and BD supplemented pens on these sampling dates, while the control treatment yielded some positive results.

The results of the statistical analysis indicated that the effect of PC was significantly different from the control treatment (OR = 0.42, *p* = 0.039), while the effect of BD was not (OR = 0.43, *p* = 0.099) when analyzed using the GEE approach given in Table 2. However, the effect of BD was meaningful and comparable to PC. When compared to the measures used in May 2021 (beginning of the sampling period), the prevalence in June 2021 and September 2021 (end of the sampling period), were significantly higher throughout the different treatment groups (*p* = 0.04, *p* = 0.01, respectively).

Since very few samples were positive for *E. coli* O157:H7 throughout the study, no statistical analysis was performed for the concentration measured by the modified MPN method. Enumeration was not performed in May and July 2021. However, in June 2021, the geometric means for the *E. coli* O157:H7 counts were 138.0 (*n* = 8), 790.0 (*n* = 4), and 3.6 (*n* = 2) MPN/g for the control, BD and PC groups, respectively. In August 2021, two control samples that tested positive had a geometric mean of 39.3 MPN/g. In September 2021, the *E. coli* O157:H7 geometric mean of counts was 4.6 (*n* = 11), 11.8 (*n* = 3), and 3.4 (*n* = 5) MPN/g for the control, BD, and PC groups, respectively.

#### 3.1.2. *Salmonella* Prevalence

**Figure 2:**
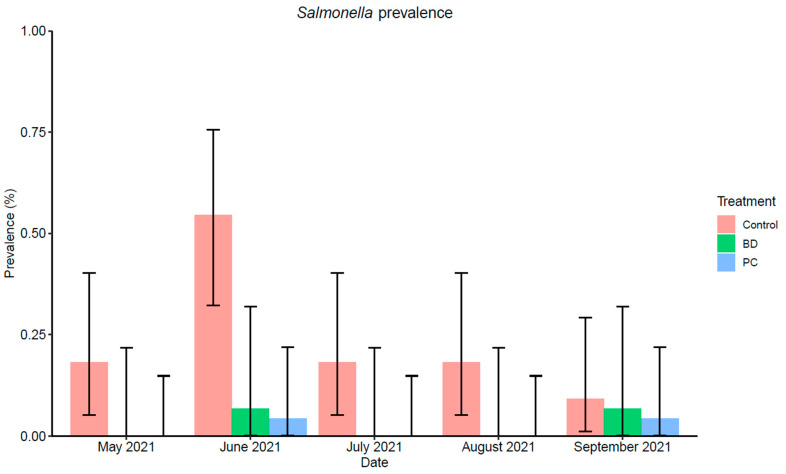
*Salmonella* prevalence (%) in fecal samples collected from the pen floors over the course of a 5-month sampling period at three different feed yards (*n* = 3 pens/feed yard). Control (May: 4/22, June: 12/22, July: 4/22, August: 4/22, September: 2/22); BD (May: 0/15, June: 1/15, July: 0/15, August: 0/15, September: 1/15); PC (May: 0/23, June: 1/23, July: 0/23, August: 0/23, September: 1/23). Error bars represent 95% confidence intervals (CI).

The average *Salmonella* prevalence in feces was 24% (26/110), 2% (2/115), and 3% (2/75) for the control, PC, and BD supplemented cattle, respectively. *Salmonella* prevalence for the two treatments and a control group over the experimental period is represented in Figure 2. Initially in May 2021, none of the fecal samples were positive for feces collected from pens housing cattle supplemented with PC and BD, while the control group had a prevalence of 18% (95% CI: 5, 40). During sampling in June 2021, the prevalence in all three groups increased, and during the consecutive two months a prevalence similar to the initial sampling dates was observed. At the end of the five-month sampling period (September 2021), the prevalence of *Salmonella* was 9% (95% CI: 1, 29), 4% (95% CI: 0, 22), and 7% (95% CI: 0, 32) for the control, PC, and BD feces samples, respectively. There was no significant difference at the end of the sampling period (*p* > 0.05), thus both DFMs were effective in keeping the *Salmonella* prevalence low when averaged over time.

The prevalence at the end of the sampling period was not significantly different for the control or treatment groups, when compared pairwise. However, a longitudinal analysis showed that the effect of PC (OR = 0.05, *p* < 0.001) and BD (OR = 0.07, *p* < 0.001) were both statistically significant over time (Table 3). Overall, the prevalence in June 2021 was significantly higher (*p* < 0.001) than the other sampling dates.

#### 3.1.3. Clostridium Perfringens Concentration

**Figure 3:**
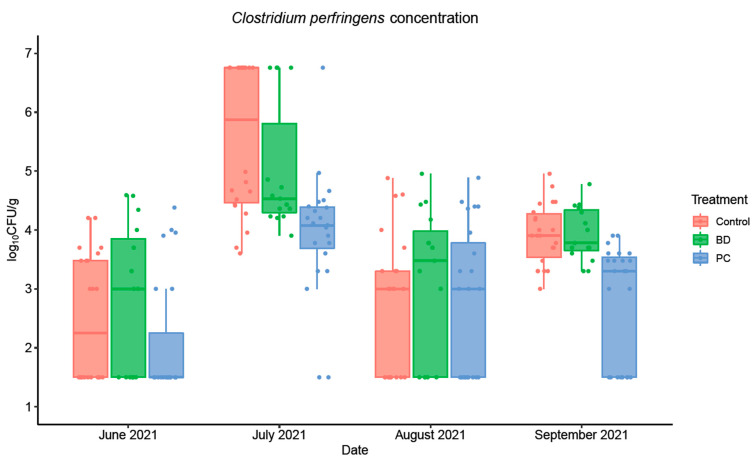
*C. perfringens* average concentration (log_10_CFU/g) in fecal samples collected from the pen floors over the course of a 4-month sampling period at three different feed yards (*n* = 3 pens/feed yard). Error bars represent 95% confidence intervals (CI).

The enumeration of *C. perfringens* in the fecal samples was conducted monthly between June and September 2021 and the data is illustrated in Figure 3. During the initial sampling in June 2021, the mean concentrations in feces were 1.77 log_10_CFU/g (95% CI: 0.95, 2.58), 0.97 log_10_CFU/g (95% CI: 0.24, 1.70), and 2.03 log_10_CFU/g (95% CI: 0.91, 3.16) for the control, PC, and BD groups, respectively. In September 2021, the concentrations for the control (3.92 log_10_CFU/g; 95% CI: 3.69, 4.15) and BD (3.93 log_10_CFU/g; 95% CI: 3.68, 4.18) treated pens were similar, but it was significantly lower in the pens treated with PC (2.27 log_10_CFU/g; 95% CI: 1.53, 3.03; *p* < 0.001). Overall, it was observed that the cattle supplemented with PC had a mean of 1.07 log_10_CFU/g reduction in fecal shedding of *C. perfringens*. The statistical analysis showed that only PC provided a significantly reduced number of *C. perfringens* throughout and at the end of the sampling period (*p* < 0.001), while BD was not significantly different from the control group (*p* > 0.05) as shown in Table 4. The effect of time was also significant during sampling in July and September 2021, indicating seasonal changes in concentrations.

**Figure 4:**
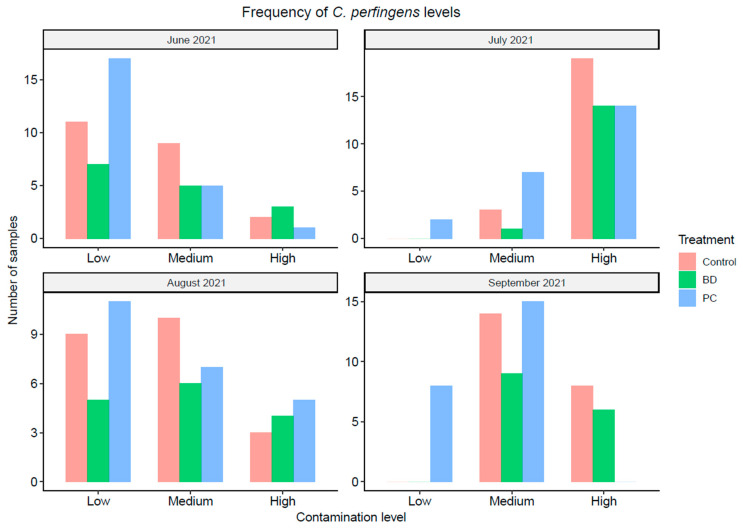
Frequency (number of samples) of *C. perfringens* contamination levels in fecal samples collected from the pen floors over a 4-month period sampling three different treatment groups. The contamination level is represented on a scale of low, medium, and high. High being > 4 log_10_CFU/g, medium being > 2 log_10_CFU/g, and low being ≤ 2 log_10_CFU/g. Each bar represents the number of samples for that treatment that fall into each contamination level category for each of the four distinct sampling dates.

The frequency of obtaining high (>4 log_10_CFU/g), medium (>2 log_10_CFU/g and ≤4 log_10_CFU/g), or low (≤2 log_10_CFU/g) counts was also considered for *C. perfringens* concentrations at four distinct sampling dates as shown in Figure 4. It was observed that the frequency of medium or low concentrations in fecal samples from the pens treated with PC was constantly higher than for the control and BD groups for all sampling dates (June through September 2021). Overall, the data highlights the performance of Probicon on controlling *C. perfringens* contamination in pen surface fecal samples by lowering the concentration in the feces, which was comparable to and often better than BD.

#### 3.1.4. Enterobacteriaceae Concentration

**Figure 5:**
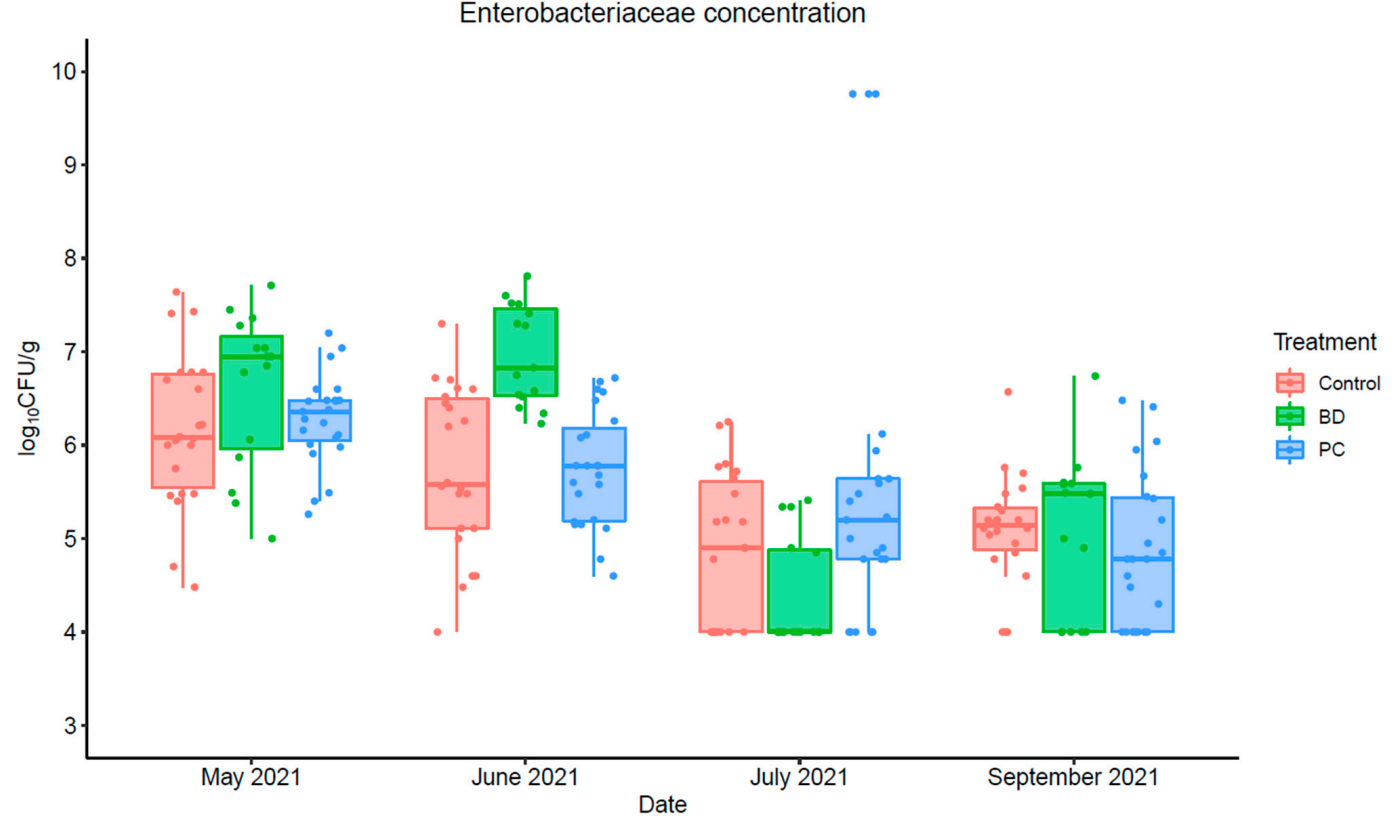
Enterobacteriaceae concentration in fecal samples collected from the pen floors over the course of 4-month sampling period at three different feed yards (*n* = 3 pens/feed yard). Error bars represent 95% confidence intervals (CI).

The enumeration of Enterobacteriaceae bacteria in fecal samples was conducted over a 4-month period between May to September 2021 as an indicator, excluding the month of August 2021. The mean counts of Enterobacteriaceae were above 4 log_10_CFU/g for all treatments throughout the entire sampling period, and achieved a minimum 1 log_10_CFU/g reduction, naturally, as shown in Figure 5. The effect of both DFM treatments were not statistically significant (*p* > 0.05) throughout the experimental period, as shown in Table 5. During the sampling period a mean reduction of 1.32 ± 0.17 log_10_CFU/g was observed for all the treatment groups. However, the counts were statistically significant during July and September 2021 (*p* < 0.01) with a decreasing trend over time (Table 5).

### 3.2. Peripheral Lymph Node Samples

#### *Salmonella* Prevalence

**Figure 6:**
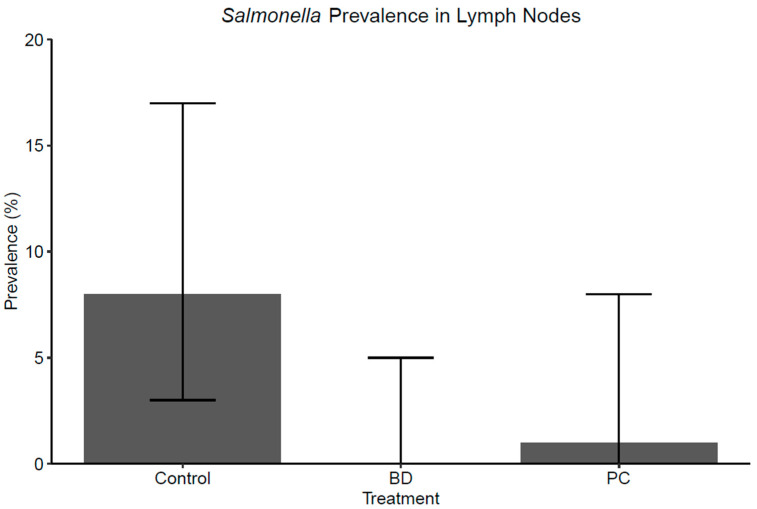
*Salmonella* prevalence (%) in bovine, subiliac, peripheral lymph node samples collected from the carcasses of the same cattle that were studied in the fecal portion of this study. The total number of positives for each treatment group, from all PLNs collected was Control: 6/74, BD: 0/72 and PC: 1/69. Error bars represent 95% confidence intervals (CI).

The lymph nodes collected from the control group cattle and from the cattle housed in pens treated with Probicon and Bovamine Defend were compared using Fisher’s exact test. The prevalence of *Salmonella* was 8.11% (6/74) in the control group, 1.45% (1/69) in the PC group, and 0% (0/72) in the BD group, as shown in Figure 6. The difference among three treatment groups were significant according to the Fisher’s test (*p* = 0.015) indicating that at least one treatment group was different from another and the prevalence for BD was significantly different from the control group (*p* = 0.028). However, the prevalence for PC was not statistically different from either the BD or the control group (*p* > 0.05). It should be noted that although Fisher’s exact test is suitable for small samples and low cell counts, the statistical power of the test was impacted by the low prevalence and limited sample size. The OR was 0.17 (95% CI: 0.02, 1.42) and the risk ratio (RR) was 0.18 (95% CI: 0.02, 1.45) when comparing the odds of detecting positive PLNs in cattle treated with PC compared to the control group, and a 39.63% (95% CI: 11.37, 50.00) prevented fraction in the population and an 82% relative risk reduction (RRR) were estimated. For BD, OR was 0.08 (95% CI: 0.00, 1.44), RR was 0.09 (95% CI: 0.00, 1.50), prevented fraction in the population was 49.32% (95% CI: 39.66, 50.00) and RR was 91%.

## 4. Discussion

In this study, the average *E. coli* O157:H7 prevalence was 20%, 10%, and 11% for control, PC, and BD groups, respectively. The cattle supplemented with Probicon had a significantly reduced prevalence (*p* < 0.05) of *E. coli* O157:H7 in pen surface fecal samples compared to the control group, although the initial prevalence was higher than the other two groups, 17.4% compared to 0% for the control group and 6.67% for the BD group. However, initial prevalence was low compared to similar previous reports within a range from 12% to 42% [42,43,44,45] and most studies sampled fecal grabs rather than pen surface material. According to a systematic review and meta-analysis by Weisener et al., a pooled OR of 0.55 (95% CI: 0.45, 0.68) with moderate heterogeneity was estimated, indicating a reduction in prevalence comparable to the results obtained in this study [44].

The average *Salmonella* prevalence in this study was 24%, 2%, and 3% for the control, PC, and BD groups, respectively. The data indicated that both DFM treatments were similarly effective in reducing prevalence, when averaged overtime, compared to no DFM supplementation, corresponding to a lower risk of environmental dissemination. Previous studies on the reduction of *Salmonella* prevalence in cattle treated with DFMs are scarce. In a similar study, Tabe et al. reported no significant difference between the fecal prevalence of naturally infected cattle treated with *L. acidophilus* (LA51) and *p. freudenreichii* (PF24) and control groups followed up for nine weeks [42]. However, they noted that DFM supplements were effective in preventing new *Salmonella* infections among the studied cohorts. Stephens et al. reported 48%, 38%, and 10% reductions in the likeliness of detecting high, medium, and low concentrations, respectively in the feces of cattle treated with BD compared to a control group [22]. Although *Salmonella* was not enumerated from pen surface fecal samples during this study, reductions in the overall prevalence are in accordance.

Although *C. perfringens* is a clinically important human and livestock pathogen [46] the effect of DFM supplements in cattle has not been widely studied. Schoster et al. and Golić et al. reported successful in vitro inhibition of *C. perfringens* by *L. helveticus*, *L. fermentum*, *Streptococcus thermophilus*, and a variety of commercial strains of *Lactobacillus* and *Bifidobacterium* as potential DFMs [47,48]. Golić et al. also reported undetectable levels of *C. perfringens* in goats after probiotic treatment and another trial in commercial broilers with a commercial DFM significantly reduced the *C. perfringens* concentration in broiler chickens [48,49]. Our results show that the novel DFM strain, *Lactobacillus salivarius* L28, can be effective in reducing the *C. perfringens* concentration in cattle feces, which would reduce the dependency on antibiotics during cattle production.

The *Salmonella* prevalence in this study was 8.11%, 1.45%, and 0% for the control, PC, and BD groups, respectively. Bovamine Defend was statistically significant from the control group (*p* < 0.05), but it should be noted that the low prevalence and small sample size impacts the statistical power of the test. In a previous study, Vipham et al. reported a RRR of 50% (RR:0.50) and 31% (0.69) for detecting positive subiliac lymph nodes in commercial feedlot cattle treated with NP51 and NP24 [31]. Furthermore, they estimated an 82% RRR (RR:0.18) in research feedlot cattle within the same study settings. Brown et al. conducted an artificial challenge test to address PLN contamination in cattle quantitatively and qualitatively using three DFM formulations containing various *Lactobacilli* and *Pediococcus acidilactici* [50]. Although the number of *Salmonella* positive PLNs from the treated cattle were numerically lower in the treatment groups (63% and 68.8% vs. 80.0%), no statistical significance was detected. However, the quantitative results indicated a significant reduction in *Salmonella* concentrations in PLNs. Therefore, the results of this study, when compared to previous studies, indicate that Probicon might offer significant reductions in *Salmonella* prevalence to mitigate ground beef contamination through PLNs.

## 5. Conclusions

The direct-fed microbials used in this study were effective in reducing the fecal shedding of *E. coli* O157:H7, *Salmonella*, and *C. perfringens* and the prevalence of *Salmonella* in PLNs, which means it may be an effective pre-harvest intervention. Furthermore, while both DFMs were effective, feces collected from the cattle housed in pens treated with Probicon had significantly less prevalence of *E. coli* O157:H7 and *Salmonella* and *Clostridium perfringens* concentration, thus it gave a better food safety outcome. The reduction of *C. perfringens* can be beneficial not only for food safety, but also for animal health in feedlot settings. While supplementing cattle with Probicon does not eliminate the fecal shedding of human and animal pathogens, it provides an effective solution to targeting multiple foodborne pathogens within a single intervention in the hope of controlling the incoming pathogen loads on harvest-ready feedlot cattle, thus lowering the risk to the consumer. Probicon also served as an effective intervention in reducing *Salmonella* prevalence in bovine PLNs, which may aid in less ground beef contamination and help respond to the public health burden. Overall, these results support the use of DFMs in a feedlot setting, but further research should be conducted on a larger scale in commercial feedlots to determine the efficacy of DFMs, specifically Probicon (L28), on the reduction of foodborne pathogens in fecal shedding and the prevalence of *Salmonella* within bovine PLNs.

## Figures and Tables

**Table 1:** Overview of the fecal sampling design with corresponding sample sizes.

Date	Treatment	Number of Samples		
		Pen 1	Pen 2	Pen 3
26-May-2021	Control	10	10	2
28-Jun-2021	Control	10	10	2
27-Jul-2021	Control	10	10	2
23-Aug-2021	Control	10	10	2
21-Sep-2021	Control	10	10	2
26-May-2021	BD	5	5	5
28-Jun-2021	BD	5	5	5
27-Jul-2021	BD	5	5	5
23-Aug-2021	BD	5	5	5
21-Sep-2021	BD	5	5	5
26-May-2021	PC	10	10	3
28-Jun-2021	PC	10	10	3
27-Jul-2021	PC	10	10	3
23-Aug-2021	PC	10	10	3
21-Sep-2021	PC	10	10	3

**Table 2:** Results of the GEE analysis for *E. coli* O157:H7 prevalence.

Term	Estimate (lnOR)	Standard Error	*p*-Value
Intercept	−1.92	0.60	0.001
Probicon	−0.88	0.43	0.039
Bovamine Defend	−0.85	0.52	0.099
June 2021	1.33	0.64	0.039
July 2021	−1.69	1.01	0.093
August 2021	−0.98	1.02	0.338
September 2021	1.67	0.66	0.012

**Table 3:** Results of the GEE analysis for *Salmonella* prevalence.

Term	Estimate (lnOR)	Standard Error	*p*-Value
Intercept	−1.64	0.31	<0.001
Probicon	−3.05	0.68	<0.001
Bovamine Defend	−2.61	0.73	<0.001
June 2021	1.76	0.43	<0.001
July 2021	0.00	0.90	1.000
August 2021	0.00	0.90	1.000
September 2021	0.00	0.70	1.000

**Table 4:** Results of the GEE analysis for *C. perfringens* concentration.

Term	Estimate	Standard Error	*p*-Value
Intercept	1.97	0.29	<0.001
Probicon	−1.08	0.28	<0.001
Bovamine Defend	0.06	0.26	0.822
July 2021	3.23	0.35	<0.001
August 2021	0.66	0.37	0.071
September 2021	1.77	0.32	<0.001

**Table 5:** Results of the GEE analysis for Enterobacteriaceae concentration.

Term	Estimate	Standard Error	*p*-Value
Intercept	6.16	0.26	<0.001
Probicon	0.12	0.27	0.65
Bovamine Defend	0.45	0.28	0.11
May 2021	−0.42	0.28	0.13
July 2021	−1.30	0.49	0.007
September 2021	−1.07	0.27	<0.001

## Data Availability

The data used to support the findings of this study can be made available by the corresponding author upon request.
